# A genomics-based systems approach towards drug repositioning for rheumatoid arthritis

**DOI:** 10.1186/s12864-016-2910-0

**Published:** 2016-08-22

**Authors:** Rong Xu, QuanQiu Wang

**Affiliations:** 1 Department of Epidemiology and Biostatistics, Institute of Computational Biology, School of Medicine, Case Western Reserve University, 2103 Cornell Road, Cleveland, 44106 OH USA; 2 ThinTek LLC, Palo Alto, 94306 USA

**Keywords:** Systems biology, Network medicine, Drug discovery, Rheumatoid arthritis, Disease genomics

## Abstract

**Background:**

Rheumatoid arthritis (RA) is a chronic autoimmune disease characterized by inflammation and destruction of synovial joints. RA affects up to 1 % of the population worldwide. Currently, there are no drugs that can cure RA or achieve sustained remission. The unknown cause of the disease represents a significant challenge in the drug development. In this study, we address this challenge by proposing an alternative drug discovery approach that integrates and reasons over genetic interrelationships between RA and other genetic diseases as well as a large amount of higher-level drug treatment data.

We first constructed a genetic disease network using disease genetics data from Genome-Wide Association Studies (GWAS). We developed a network-based ranking algorithm to prioritize diseases genetically-related to RA (RA-related diseases). We then developed a drug prioritization algorithm to reposition drugs from RA-related diseases to treat RA.

**Results:**

Our algorithm found 74 of the 80 FDA-approved RA drugs and ranked them highly (recall: 0.925, median ranking: 8.93 %), demonstrating the validity of our strategy. When compared to a study that used GWAS data to directly connect RA-associated genes to drug targets (“direct genetics-based” approach), our algorithm (“indirect genetics-based”) achieved a comparable overall performance, but complementary precision and recall in retrospective validation (precision: 0.22, recall: 0.36; F1: 0.27 vs. precision: 0.74, recall: 0.16; F1: 0.28). Our approach performed significantly better in novel predictions when evaluated using 165 not-yet-FDA-approved RA drugs (precision: 0.46, recall: 0.50; F1: 0.47 vs. precision: 0.40, recall: 0.006; F1: 0.01).

**Conclusions:**

In summary, although the fundamental pathophysiological mechanisms remain uncharacterized, our proposed computation-based drug discovery approach to analyzing genetic and treatment interrelationships among thousands of diseases and drugs can facilitate the discovery of innovative drugs for treating RA.

## Background

RA is a chronic inheritable autoimmune disease characterized by inflammation and destruction of synovial joints. RA affects up to 1 % of the population worldwide [1]. The cause of RA remains unknown, with multiple genetic and environmental factors involved [2]. Currently, there is no drug that can cure RA or achieve sustained remission.

Disease genetics can lead to the identification of novel drug treatments. Over the past decade, genome-wide association studies (GWAS) have robustly identified genetic risk loci linked to many complex diseases, including 100 risk loci for RA [3], and have provided valuable biological insights into many common diseases [4]. Several recent studies have indicated that disease genetics identified by GWAS may lead to translational opportunities for drug discovery [5–11]. For example, a recent study showed that RA risk loci identified through meta-analysis of GWAS data provided therapeutic opportunities for the repositioning of existing drugs for the treatment of RA [7]. To capitalize on complex human disease genetics identified through GWAS, the National Center for Advancing Translational Science (NCATS) was established to use genomic information to determine whether drugs approved to treat one disease could be effective in treating others [8]. However, significant challenges exist in directly translating disease-associated genetic variants identified by GWAS into novel therapeutics [4].

**Fig. 1:**
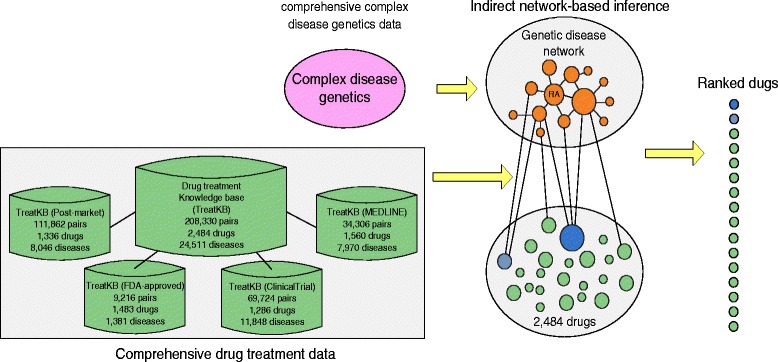
Indirect disease genetics-based drug repositioning approach

Here, we propose an alternative drug discovery approach by combining lower-level disease genetics identified by GWAS with higher-level drug treatment databases that we recently constructed [12–14]. We hypothesize that the genetic overlap among diseases often reflects pathophysiological overlap. Though the majority of such shared pathophysiological features may remain unknown (i.e. RA biology), treatment insights from one disease may be used to inform our knowledge of others and potentiate their treatments. Instead of directly inferring drug targets from disease genetics as previous approaches [5–7, 15], we use diseases that share high degrees of genetic commonalities with RA (RA-related diseases) as the starting point for discovering new drug treatments for RA (Fig. 1). For example, if a drug treats many RA-related diseases, then this drug is more likely to be a promising candidate to treat RA than a drug that treats few RA-related diseases. Compared to the traditional disease genetics-based or target-based drug discovery, we can put such inference into practice without precise knowledge of disease biology or drug mechanisms.

Computational drug repositioning approaches can be classified as either drug-based or disease-based [16, 17]. Drug-based approaches leverage upon known drug molecular structures or functions such as chemical structure and properties, molecular docking, gene expression, drug treatments, and drug side effects [18–22]. It was recognized that drug screening based on existing drugs (“drug-based”) might fail to identify new therapeutic mechanisms [23]. On the other hand, disease-based approaches put less emphasis on existing drugs and focus more on disease mechanisms or interrelationships among diseases, therefore have potential in discovering truly innovative drugs. Disease-based approaches have used disease-related data ranging from genome [19, 20] to phenome [24–27]. While existing drug repositioning systems often used well-established computational or statistical methods, including regression/classification, machine learning, network analysis, and text mining [17], they differ in the datasets included in the systems and how heterogeneous data are integrated.

The keys to our drug repositioning system include both the unique datasets included in the system as well as innovative approaches to integrating various disease- and drug-related data. One of the key components of our system is four large-scale drug-disease treatment relationship knowledge bases (TreatKBs) that we recently constructed from multiple heterogeneous and complementary data resources using advanced computational techniques including natural language processing, text mining and data mining [12–14]. The four TreatKB include 9,216 drug-disease treatment pairs extracted from FDA drug labels, 111,862 pairs extracted from the FDA Adverse Event Reporting System (FAERS), a database supporting the FDA’s post-marketing drug safety surveillance, 34,306 pairs extracted from 22 million published biomedical literature abstracts, and 69,724 pairs extracted from 171,805 clinical trials. All together, TreatKB contains 208,330 drug-disease treatment pairs for 2484 drugs and 24,511 diseases. In addition, we applied a novel signal prioritization algorithm that we recently developed [25], which first identifies diseases that are genetically related to RA and then prioritizes drugs based on the relevance of their associated diseases to RA.

## Methods

**Fig. 2:**
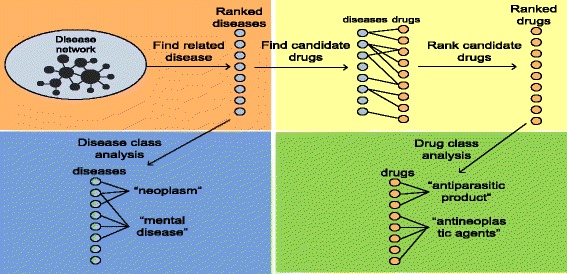
The overall experiment flow chart

The experiment framework is depicted in Fig. 2 and consists of four steps: (1) we constructed a genetic disease network (GDN) using disease-gene associations from GWAS. We developed a network-based ranking algorithm to find diseases that shared high degrees of genetic commonality with RA; (2) we analyzed disease classes that were highly associated with RA in order to evaluate the disease-ranking algorithm and to gain insight into RA-related diseases; (3) we developed a drug prioritization algorithm to systematically reposition drugs from RA-related diseases to treat RA. We retrospectively validated the algorithm using 80 FDA-approved RA drugs. We evaluated our algorithm in novel predictions using 165 not-yet-approved RA drugs. We compared our approach (“indirect disease genetics-based”) to Okada’s study [7] (“direct disease genetics-based”) in both retrospective validation and novel predictions; and (4) we examined drug classes that were highly enriched among top-ranked drug candidates.

### Find and analyze RA-related diseases

#### Construct genetic disease network (GDN)

We constructed a GDN using disease-gene associations from the Catalog of Published Genome-Wide Association Studies from the US National Human Genome Research Institute (NHGRI), which is an exhaustive source containing the descriptions of disease- and trait-associated single nucleotide polymorphisms (SNPs) from published GWAS data [28]. We obtained a total of 22,470 disease/trait-gene pairs, representing 881 diseases/traits and 8,689 genes.

On GDN, two diseases were connected if their associated genes overlapped. The edge weights were determined by the cosine similarity coefficients of disease-associated genes [29]. We also experimented other similarity measures such as Jaccard similarity coefficient and overlap. Since some diseases do not share genes directly but their associated genes may interact or participate in the same pathways, we also investigated alternative approaches to connect diseases on the networks. We connected two diseases if their associated genes (proteins) interact or participate in the same pathways using data from the STRING database [30]. Experimental results showed that connecting diseases based on cosine similarity of their associated genes performed best. GDN comprised of 882 disease nodes and 200,758 edges.

#### Apply network-based ranking algorithm to find RA-related diseases from GDN

We have recently developed network-based ranking algorithms to prioritize genes for a given disease [26, 31] or to prioritize diseases for a given microbial metabolite [32]. The iterative network-based ranking algorithm is defined as: *p*^*t*+1^=(1−*γ*)*Mp*^*t*^+*γp*^0^, wherein *M* is the column-normalized adjacency matrix of GDN, *γ* is a preset probability of restarting from the initial seed node (*γ*=0.1 in this study), and *p*^*t*^ is a vector in which the *i*_*th*_ element holds the normalized ranking score of disease *i* at *t*_*th*_ iteration. The initial probability vector *p*^0^ contains only RA, with a probability of 1.0. The iterative ranking algorithm was run until it converges, meaning that the change between *p*^*t*+1^ and *p*^*t*^ is less than 10^−6^. Diseases are ranked according to values in the steady-state probability vector *p*^*t*^.

#### Analyze RA-related diseases

To better understand top-ranked diseases as well as to test the network construction and ranking algorithms, we examined the distributions of disease classes among RA-related diseases at different ranking cutoffs. We classified diseases based on the 10th revision of the International Statistical Classification of Diseases and Related Health Problems (ICD10) [33]. The ICD10 includes 22 highest-level disease classes (or chapters) such as “Neoplasms” and “Diseases of the nervous system.” During our experiments, we found that only six disease classes were well represented in the GWAS catalog: *immune diseases*, *autoimmune diseases*, *cardiovascular diseases*,*metabolic disorders*, *mental or psychiatric disorders*, and *neoplasms*. The two disease classes: *immune diseases* and *autoimmune diseases*, served as positive controls because RA is an autoimmune disease (and immune disease) and is expected to be related to other immune diseases. We retrieved a ranked list of diseases from GDN using RA. We calculated the percentage of these six disease classes among ranked diseases at 10 different ranking cutoffs (top 10 %, 20 %,... 100 %).

### Reposition drugs based on RA-related diseases

#### Drug repositioning algorithm

We have recently developed a drug prioritization approach to systematically reposition drugs that treat RA-related diseases to treat RA [25]. The algorithm is based on the assumption that if a drug treats many top-ranked RA-related diseases, it will rank higher than another drug that treats one or two lower-ranked diseases. We first ranked drugs based on the number of RA-related diseases that they could treat as well as the ranking scores of these diseases. The drug prioritization algorithm is defined as: $R_{drug}=\sum \limits _{i=1}^{n} R\_disease\_i$, wherein *n* is the number of RA-related diseases that a drug can treat and *R*_*disease*_*i* is the disease ranking score (output from the network-based disease ranking algorithm).

#### *De novo* validation using known RA drugs and comparison

We evaluated our algorithm using a total of 80 FDA-approved RA drugs. This list of drugs were manually compiled from FDA drug label and several authoritative medical-related websites such as webMD.com and drugs.com. Our evaluation was a *de-novo* validation scheme, since RA and its associated drug treatment pairs were removed from the inputs to the repositioning algorithm. The standard precision, recall, and F1 measures were used.

We compared our study to Okada’s study [7] in predicting both FDA-approved RA drugs as well as novel drugs (discussed in the next section). In Okada’s study, the authors first prioritized 98 RA risk genes, from which a total of 27 drug target genes of approved RA drugs were identified. These 27 drug target genes were then connected to 19 drugs. Among these 19 drugs, 14 were FDA-approved RA drugs and 5 were novel predictions. We could not compare our approach to one of the state-of-art drug repositioning systems, PREDICT [24], since it does not include RA. PREDICT used disease-disease and drug-drug similarities from multiple data resources to construct a classifier to determine treatment associations between 593 drugs and 313 diseases, the majority of which are Mendelian diseases. One of PREDICT’s limitations is that it can only infer new connections between the 593 drugs and 313 diseases included in the system.

#### Evaluate Novel predications

The fact that a drug repositioning algorithm has worked well in ranking known RA drugs (retrospective validation) does not imply that it will work equally well in novel drug prediction; therefore, evaluation of novel prediction capability is critical for any drug repositioning algorithm. Instead of manually searching literature or clinical trials for evidence supporting novel predictions, we automated this process using the drug-disease treatment knowledge bases that we constructed from over 22 million biomedical literature records and from 172,888 clinical trials. We extracted a total of 162 RA drugs from published biomedical literature, including 49 FDA-approved RA drugs and 113 novel drugs. We extracted a total of 103 RA drugs from clinical trials, including 37 FDA-approved RA drugs and 66 novel drugs. Combining RA drugs from these two resources, we obtained a total of 165 novel RA drugs after FDA-approved drugs were removed. We evaluated the performance of our algorithm in novel prediction using these 165 drugs. We calculated precisions, recalls, and F1 measures at different ranking cutoffs. We compared our study to Okada’s study in novel predictions using the same evaluation dataset.

### Analyze repositioned drug candidates

To better understand top-ranked repositioned drug candidates, we examined which classes of drugs (as defined by the Anatomical Therapeutic Chemical (ATC) classification system [34]) were enriched. Drug classes enriched among top-ranked drug candidates could provide insights into the underlying mechanisms of action of drug candidates within those classes. The ATC system consists of 13 first-level codes, 94 second-level codes, 267 third-level codes, 882 fourth-level codes, and 4580 fifth-level codes, which are individual drugs. We experimented the drug classification using different levels of ATC codes and found that third level ATC codes gave sufficient granularity. We calculated percentages of drug classes associated with the top 10 % of ranked drugs and compared them to those for all drugs. We identified drug classes that showed at least a 2-fold enrichment.

## Results

### Evaluate network construction and disease ranking algorithms and analyze RA-related diseases

**Fig. 3:**
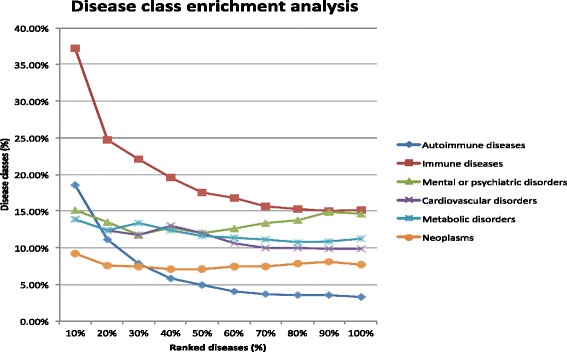
Disease class distribution among RA-related diseases at 10 ranking cutoffs

We retrieved a ranked list of 842 diseases from the GDN using RA as the input. Two classes were highly enriched among top-ranked diseases: *autoimmune diseases* and *immune diseases* (Fig. 3). Among top 10 % of retrieved diseases, 18.6 % were autoimmune diseases, representing a significant 5.59-fold enrichment as compared to the 3.33 % among all retrieved diseases. We also observed a significant enrichment for immune diseases. Among the top 10 % of diseases, 37.21 % were immune diseases, representing a significant 2.45-fold enrichment as compared to 15.20 % among all retrieved diseases. These results were expected because RA itself is an immune disease as well as an autoimmune disease. These results demonstrate the validity of both network construction and disease ranking algorithms. The other four disease classes were not significantly enriched.

### Retrospective validation with 80 FDA-approved RA drugs

#### Drug repositioning using the combined TreatKB has better performance than individual TreatKBs

We validated our drug repositioning algorithm using 80 FDA approved RA drugs. Since the drug treatment knowledge bases (TreatKBs) were constructed from different data resources using different computational methods, we evaluated which TreatKBs performs better in drug repositioning for RA. We calculated recalls, mean, and median rankings of these FDA-approved RA drugs when the four different TreatKBs were used separately or combined (Table 1). When the TreatKB derived from FDA drug labeling (‘FDA-approved’) was used, we achieved a recall of 0.825, an average ranking of 36.58 %, and a median ranking of 34.46 %. We achieved significantly better rankings when the two TreatKBs that were constructed from post-marketing FAERS (recall: 0.775, mean ranking: 19.02 %, and median ranking: 8.53 %) and from the biomedical literature (recall: 0.663, mean ranking: 29.69 %, and median ranking: 19.17 %) were used, respectively. Significantly, when the combined TreatKB was used, we achieved a recall of 0.925 (74 out of 80 RA drugs), a mean ranking of 20.0 %, and a median ranking of 8.93 %. The significantly improved recall and rankings demonstrate the critical importance of a comprehensive drug treatment knowledge base in drug repositioning tasks. In comparison, such high rankings were not evident in our analysis for randomly selected FDA-approved drugs (49.76 % for mean ranking and 44.65 % for median ranking).

**Fig. 4:**
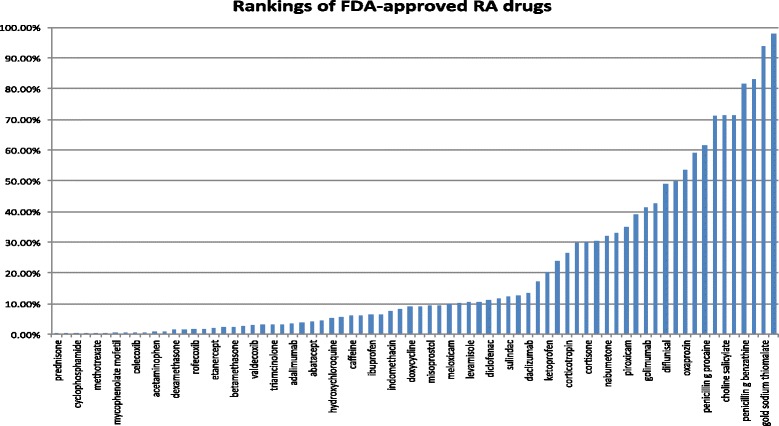
Rankings of (74 out of 80) FDA-approved RA drugs among 2484 drugs. The combined TreatKB was used

As shown in Table 1, there is a significant difference between the median ranking of 8.93 % and the mean ranking of 20.05 %, demonstrating a skewed ranking distribution of these RA drugs. Fig. 4 shows that the rankings of RA drugs varied greatly from 0.04 % (prednisone) to 97.83 % (salicylamide). These results indicate that not all RA drugs can be discovered based on disease genetics.

**Table 1:** Recalls, mean, and median rankings of 80 FDA-approved RA drugs when four TreatKBs were used separately and in tandem

TreatKB	Recall	Mean ranking	Median ranking
FDA-approved	0.825	36.58 %	34.46 %
Post-market	0.775	19.02 %	8.53 %
Clinical trials	0.750	31.73 %	29.24 %
Literature	0.663	29.69 %	19.17 %
***Combined***	***0.925***	***20.06 %***	***8.93 %***

The best performance was achieved when four TreatKBs were combined (bold data)

### Our approach has comparable overall performance but complementary precision and recall as compared to a “direct genetics-based” approach

**Fig. 5:**
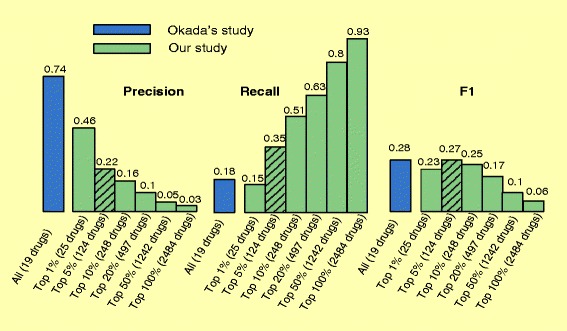
The performance (precisions, recalls, and F1s) of our algorithm at six different cutoffs (top 1 %, 5 %, 10 %, 20 %, 50 % and 100 % [all 2484 drugs]) when evaluated using 80 FDA-approved RA drugs. For comparison, Okada’s study is shown in blue

As shown in Fig. 5, our drug repositioning algorithm proved effective in ranking FDA-approved RA drugs at the top: the precision was 0.46 for top 25 drugs (top 1 %), which represents a significant 14.3-fold enrichment as compared to the 0.03 for all 2484 drugs (top 100 %). The best overall performance was achieved at a cutoff of 5 % (top 124 drugs): a precision of 0.22, a recall of 0.35, and an F1 of 0.27 were achieved.

We compared our study to Okada’s study in prioritizing known RA drugs. When evaluated using the 80 FDA-approved RA drugs, Okada’s study achieved a recall of 0.175, a precision of 0.736 and an F1 of 0.28. Our algorithm, at a 5 % cutoff (top 124 drugs), achieved a precision of 0.22, a recall of 0.35, and an F1 of 0.27 (Fig. 5). While Okada’s study achieved a higher precision, our study achieved a higher recall, indicating that these two approaches are largely complementary.

### Evaluation of novel predictions using 165 novel RA drugs

**Fig. 6:**
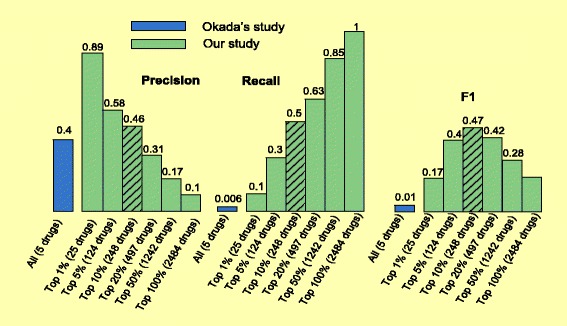
The performance (precisions, recalls and F1s) of our algorithm at six different cutoffs (top 1 %, 5 %, 10 %, 20 %, 50 % and 100 % [all 2484 drugs]) when evaluated using 165 novel RA drugs. For comparison, Okada’s study is shown in blue

We evaluated our algorithm in novel prediction. As shown in Fig. 6, our drug repositioning algorithm proved effective in prioritizing novel RA drugs, achieving a precision of 0.89 for the top 25 drugs (top 1 %), which represents a significant 8.9-fold enrichment as compared to the 0.1 for all 2484 drugs. The best overall performance in novel prediction was achieved at a cutoff of 10 % (top 248 drugs): a precision of 0.46, a recall of 0.50, and an F1 of 0.47.

Okada’s study made a total of five novel predictions (auraofin, certolizumab pegol, lguratimod, tacrolimus, and temsirolimus). Among these five drugs, two drugs (certolizumab pegol, and tacrolimus) appeared in the evaluation dataset: both certolizumab pegol and tacrolimus currently are in active clinical trials. Therefore, Okada’s study has a precision of 0.40, a recall of 0.006 (2 out of 165 novel RA drugs), and an overall F1 of 0.01 when evaluated using the set of 165 drugs (Fig. 6). The best performance for our system was achieved at a cutoff of 10 % (top 248 drugs): a precision of 0.46, a recall of 0.50, and an F1 of 0.47, representing a 47-fold increase in F1 as compared to the F1 of 0.01 in Okada’s study. The drug tacrolimus from Okada’s study was ranked at top 0.76 % position (top 19 among all 2484 drugs) by our algorithm. In summary, we show that our repositioning strategy has comparable overall performance (yet complementary precision and recall) to Okada’s study in retrospective validation using the FDA-approved RA drugs. However, our algorithm has performed significantly better in finding novel RA drugs, and therefore has greater potential in the task of discovering innovative drug treatments for RA.

### Drug categories for top-ranked drug candidates offer insight into the underlying mechanisms of drug actions

**Fig. 7:**
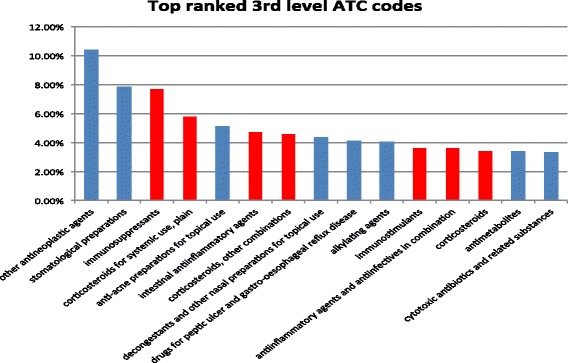
Top 15 third-level ATC codes for top 10 % repositioned drug candidates that show at least 100 % enrichment as compared to percentages of the same ATC codes for all drugs. Both immune- and inflammation-related ATC codes were highlighted as *red*

Figure 7 shows 15 third-level ATC codes that showed at least 100 % enrichment for the top 10 % ranked drug candidates as compared all drugs. As shown in the Fig. 7, 7 out of these 15 ATC codes are related to immune reaction modulation and inflammation, including Immunosuppressants, corticosteroids, and anti-inflammatory agents. In the disease class enrichment analysis, we also showed that both immune and autoimmune diseases were highly enriched among top-ranked RA-related diseases. Both analyses are consistent with the fact that immune deregulation is implicated in the development of RA. This result also demonstrates that common pathophysiological mechanisms are shared among RA-related diseases. Intriguingly, anticancer drugs are the most highly enriched drug class (Fig. 7). This is consistent with findings from Okada’s study indicating that SNPs in RA risk genes overlapped with somatic mutation genes in cancers. In particular, some genes involved in the development of hematological cancer were implicated. This is also consistent with the fact that several approved RA drugs (for example, rituximab) were initially developed for cancer treatment and subsequently repurposed for the treatment of RA. It will be interesting to further investigate top-ranked anticancer drugs in the treatment of RA experimentally.

## Discussion and conclusions

In this study, we prioritized a total of 2484 drugs in terms of their relevance in the potential treatment of RA. While previous studies demonstrated that directly linking disease-associated genes from GWAS data to drug targets can lead to novel drug discovery, our study provides an alternative strategy to capitalize on complex human 5 genetics and comprehensive drug treatment data for other diseases for the discovery of innovative drug treatments for RA. Our algorithm retrieved 74 out of 80 FDA-approved RA drugs and ranked those drugs highly, demonstrating the validity of our approach. In addition, our algorithm proved effective in predicting innovative drugs for RA. Nonetheless, our study can be significantly improved upon in the future.

Our current study was restricted by the limited number of diseases (881 diseases) in the GWAS catalog, even though TreatKB includes 24,511 diseases. With new studies being continually added to the GWAS catalog, as well as new disease-gene associations increasingly being revealed by next-generation sequencing studies, additional drug repositioning opportunities will arise through human genetic analysis. Additionally, disease genetics from rare Mendelian disorders represents another valuable source of novel drug targets and may lead to surprising and novel drug discovery opportunities [15, 35]. Another rich resource for knowledge of human disease genetics is computation-based candidate disease gene prediction. Computational disease gene prediction aims to find new disease-gene associations through integrative computational analysis of known data of diseases, genes, functional protein interactions, gene expression, and the biomedical literature, among many others [36]. We recently showed that computationally-predicted disease genetics can lead to novel drug discovery [26, 31]. In the future, we will combine comprehensive disease genetics data from all three of the above-described sources with a novel computational strategy to find new drug treatments for RA.

This study focuses on disease genetics-based drug repositioning. Additional invaluable resources such as other disease-related data (i.e. disease phenotypic data or gene expression data) and drug-related data (i.e. drug side effects, drug chemical structure, and gene expression) can be incorporated into the currently proposed algorithm to further improve performance. Integrating and reasoning over such complex biological data poses a significant challenge that bears future investigation.
